# The Integration of Metagenomics and Chemical Physical Techniques Biodecoded the Buried Traces of the Biodeteriogens of Parchment Purple Spots

**DOI:** 10.3389/fmicb.2020.598945

**Published:** 2020-12-21

**Authors:** Nicoletta Perini, Fulvio Mercuri, Silvia Orlanducci, Maria Cristina Thaller, Luciana Migliore

**Affiliations:** ^1^Department of Biology, Tor Vergata University, Rome, Italy; ^2^Department of Industrial Engineering, Tor Vergata University, Rome, Italy; ^3^Department of Chemical Science and Technology, Tor Vergata University, Rome, Italy

**Keywords:** NGS, RAMAN, LTA, parchment, halophiles

## Abstract

Ancient parchments record an immense part of our cultural heritage, having been used as the main written support material for centuries. Parchment easily undergoes biodeterioration, whose main signs are the so-called *purple spots*, which often lead to detachment of the superficial written layer. Up to recent years, several studies have been analyzing damaged parchments from different world’s archives, trying to trace back the culprit of the purple spots. However, standard cultivation and early molecular techniques have been demonstrated to be unsuccessful, leading the parchment damage issue remaining unsolved for many years. Nowadays, some studies have explored the parchment biodeterioration dynamics by adopting a multidisciplinary approach combining standard microbiological methods with high-throughput molecular, chemical and physical techniques. This approach allowed an unprecedented level of knowledge on the complex dynamics of parchment biodeterioration. This mini review discusses the application of the combination of basic and high-throughput techniques to study historical parchments, highlighting the strengths and weaknesses of this approach. In particular, it focuses on how metagenomics has been paramount for the unequivocal identification of the microbial main actors of parchment biodeterioration and their dynamics, but also on how metagenomics may suffer the distortion inflict by the historical perspective on the analysis of ancient specimens. As a whole, this mini review aims to describe the *scenario* of information on parchment biodeterioration obtained so far by using the integration of metagenomic with recent chemical (Raman spectroscopy) and physical (Light Transmission Analysis) approaches, which might have key implications in the preservation of many ancient documents.

## Introduction

The preservation of cultural heritage is one of the major challenges of today’s society, because of the fundamental right of future generations to inherit it. Writing has always been one of the most effective communicative ways of transmission of the cultural identity and memory; hence, ancient manuscripts represent milestones of cultural uniqueness of a country. For over two millennia, the most used writing material was parchment, a semi-solid collagen matrix, obtained from animal skins (Migliore et al., 2016). Hence, parchments preserved our history and cultural identity; this justifies the development of tools to ensure their conservation. Unfortunately, over centuries many events have caused huge losses of the world’s memories: wars, natural disasters (e.g., earthquakes or floods; van der Hoeven and van Albada, 1996), or living organisms, which colonize and deteriorate cultural heritage objects.

Biodeterioration has been longish neglected, being physical and chemical processes regarded as dominant factors of decay. In the last decades, this dogma has been countered: it is now generally agreed that microorganisms, besides esthetical damages, cause acid corrosion, enzymatic degradation, and mechanical attack (Sterflinger and Piñar, 2013). Biodeterioration is among the main causes of hide products degradation, including parchments (Caneva et al., 2007). Due to their animal origin, hide products are mainly composed of collagen, an appetizing source of nutrients for microbes, that may thrive on amino acids. Since ancient times, in the Southern regions (e.g., Italy) rawhides were treated with sea-salt to prevent skin decay (Migliore et al., 2017). Diffusion models revealed that during brining, NaCl ions gradually moves from the flesh toward the hair side (Hernàndez et al., 2008), making parchment an extreme salty environment, prohibitive for the great majority of microbes (Bailey, 2003).

Although rawhides were initially preserved in salt (Tanasi, 2002), ancient parchments often underwent microbial attack, nowadays testified by the signs known as *purple spots*, often leading to the detachment of the superficial (written) layer, and loss of historical content. Purple spots may affect the entire parchment surface, but a “respect zone” around the inks (Kolar et al., 2008; Vendittozzi, 2015). Interestingly, present-day rawhide commercial products often show signs of biodeterioration, similar to purple spots, known as *red heat deterioration* (Reed, 1972; Perini et al., 2019).

## The Causes of Purple Spots

Over decades, authors have analyzed several aspects of damaged parchments from different world’s archives. Investigations were carried out by different approaches. Culture-dependent techniques demonstrated totally ineffective in cultivating purple stain producing microbes directly from purple damaged specimens (Reed, 1972). Standard molecular and culture-independent techniques were ineffective in univocally defining the main actors of the damage, although including DNA fingerprinting by Denaturing Gradient Gel Electrophoresis, DGGE, and a 16S rDNA clone library from a pool of damaged samples (Piñar et al., 2015). Additionally, these methods are time-consuming and prone to underestimate the microbial number and diversity in the samples.

Over time, the damage was attributed to different biodeteriogens: *Serratia marcescens* (Marconi, 1976), *Streptomyces* (Karbowska-Berent and Strzelczyk, 2000), and *Saccharopolyspora* (Piñar et al., 2015). Anyway, both a common causative agent and the colonization dynamics remained elusive.

Nowadays, the availability of Next Generation Sequencing (NGS) proved revolutionary, simultaneously reducing costs, time-consuming, and the scale of genome characterization. NGS allows also to study specific genome regions and to determine community taxonomic analysis. It is therefore utilized in many research fields, including Biocodicology (Fiddyment et al., 2019), aimed to unlock biological signals, including ancient DNAs associated to the parchment, providing a deeper understanding of the artworks. Metagenomic techniques have been useful to shed light on the animal origin of the skins of archeological parchment fragments (Teasdale et al., 2015; Shepherd et al., 2019; Anava et al., 2020; Piñar et al., 2020) or to get the enumeration of different kind of parchment colonizers (Piñar et al., 2020). In the last years, metagenomics demonstrated to be pivotal in finding the causative agents and the colonization dynamics leading to parchment biodeterioration. Obviously, the analytical and bioinformatics approaches are equally critical for a proper management and interpretation of NGS data (Koboldt et al., 2013).

**FIGURE 1:**
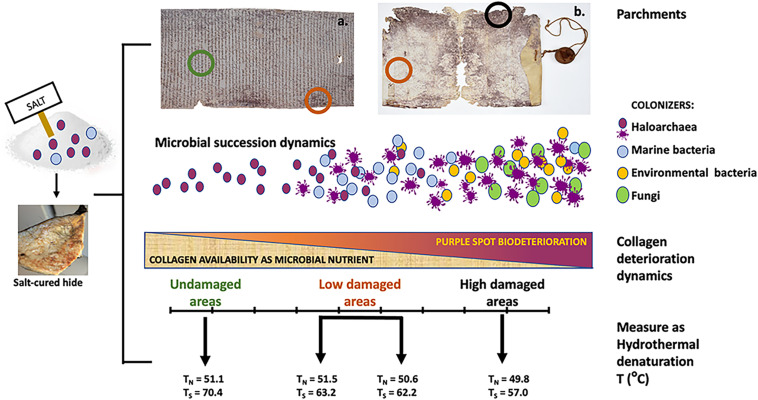
Parchment colonization model [from Migliore et al. (2017, 2019)]. The two parchments, both from the Vatican Apostolic Archives show different degree of deterioration, being the examples of the extremes of a biodeteriorative *continuum*: *left*, *A*.*A*. *Arm*. *I–XVIII 3,328, Archivum Arcis*, a well preserved parchment roll with very limited bioderiorated areas; *right*, *Faldone Patrizi A 19*, a highly damaged parchment with both limited and severe biodeterioration areas, and loss of entire parts. These different degradative output is caused by an ecological heterotrophic succession, in this case imputable to different microbial types. The early microbial colonization of the hides depends upon the salt-curing, preliminary to all the parchment production treatments. In marine salt both haloarchaea and marine bacteria are present, coming from the natural microbial communities in the evaporation ponds of salt-fields. These microbes can survive even long times in the deepest layers of the hides and reanimate at the suitable environmental conditions (humidity and high salinity). Hence, the succession starts with the haloarchaea, able to get supplementary energy by bacteriorhodopsin which convert light into chemical energy. They trigger the attack to collagen, being amino acids (particularly arginine and aspartate) their preferential food. When humidity increases and salinity decreases, they collapse, giving rise to the purple spots. At these humidity and salinity conditions marine bacteria (Gammaproteobacteria) can grow, consuming both the already attacked collagen matrix and the halobacterial debris, only leaving the purple stain. Then, on the damaged (and more readily available) parchment collagen matrix a variety of environmental available microbes can grow; the more they are available in the environment, the more they will be represented in the degradative microbial community, as in the case of the ubiquitous Actinobacteria. At this stage also fungi are recruited, able to complete the degradative process, natural final aim of every ecological heterotrophic succession. Hence, as reported in the bar (*middle of the figure*), the ongoing catabolic activities of the microbial degradative community progressively reduces the available collagen, up to its complete degradation, as happened in the lacking parts of the *Faldone Patrizi* parchment. The degradative process has been measured by light transmission analysis (*lower section of the figure*), which revealed the kind and entity of the damage suffered by the parchment collagen, as temperature of hydrothermal degradation of the two main collagen population: the less stable collagen, classified as *native* (N) and the more stable, the so called *stabilized* (S), typical of the fibers sheath and characterized by a larger thermal stability. These temperatures and the micrographic photos concurrently taken (not shown), showed a progressive damage along the degradative succession: in the undamaged parchment, a diffused matrix encompassing the more robust fibers was found; as the process of collagen degradation goes on, the matrix is completely absent and only the more robust fibers can be found; then, at the end of the degradative process the more robust fibers are completely absent and only the matrix can be found, indicating the conversion of the more organized fibers into a less organized and unstable diffused matrix. This is confirmed by the large temperature change in Ts, revealing the relevant amount of damage produced in the collagen S. This heavy damage in collagen S is concomitant to the presence of the second-phase bacterial colonizers: for example, Actinobacteria are able to deeply penetrate into the collagen structure and damage even the more robust fibers, by a slow but constant action.

The NGS-based metagenomic analysis has been firstly utilized in Biocodicology by Migliore et al. (2016) to solve the issues of an ancient roll biodeterioration, and then this approach provided a definitive insight into the purple spot causes. The analysis of the whole biological information stored in the roll allowed to bring to light the traces of microbes that inhabited the documents over centuries. These NGS-based studies on ancient parchments provided a deeper insight into the microbial communities colonizing the damaged vs. the undamaged areas of the document, depicting the “core microbiome” in each set of samples. The dominant group in both sets of samples was composed by environmental, human/animal associated bacteria, with Actinobacteria (e.g., Pseudonocardiales) being the prevalent class, mainly abundant in undamaged areas; their presence had been previously reported (Piñar et al., 2015). Moreover, NGS revealed the presence of halotolerant (e.g., Vibrionales) marine microorganisms, only or mainly in the damaged areas (Migliore et al., 2016, 2017; see Figure 1). All of these, however, could not explain the stain of the purple spots. A further hint came from the concurrent analysis of the same roll with RAMAN spectroscopy, performed both directly and on chemically extracted pigments, which detected the traces of the haloarchaea presence in the parchment (Migliore et al., 2016, 2017). RAMAN spectroscopy is a rapid and non-destructive tool that provides structural information on both organic and inorganic compounds. It was extensively used in cultural heritage to identify pigments, inks, minerals, and substrata and, in the last 10 years, successfully applied to study microorganisms and microbial pigments (Jehlička et al., 2014).

Parallelly, another up-dated physical technique, the light transmission analysis (LTA) was used to evaluate the kind and entity of the damage to native collagen. The LTA of collagen-based materials is a recently developed technique to quantitatively assess the state of collagen integrity, measuring its chemical stability trough the hydrothermal denaturation temperature (Migliore et al., 2017; Cicero et al., 2019). Under this micro-destructive analysis, some parchment fibers are heat-jellified in aqueous solution, while recording the signal generated by a beam of light passing through the sample. The signal variations during the transformation process are converted into a denaturation curve as a function of temperature, characterized by the presence of one or more peaks, each defining the denaturation temperature and therefore the integrity of the collagen population. The number and width of peaks in the denaturation curve, provide semi-quantitative information on the chemical stability of the collagen populations, highlighting inhomogeneity due to intrinsic features and/or colonizer activity. The higher the temperature corresponding to the peak position, the higher the chemical stability. Finally, the LTA experimental apparatus is integrated with a long-working-distance microscopy system, to record images in polarized light, documenting what happens during denaturation (i.e., shrinkage or loss of birefringence of the fibrous tissue), and correlating to specific features of the LTA curve (Cicero et al., 2019).

The LTA analysis on purple spotted roll, was performed by comparing the results obtained from the areas showing evident microbiological damage (purple spots) with others from intact parts of the same document. This allowed to highlight the pinpointed nature of the microbial attack, which changes the target collagen populations along the biodegradative process, determining the degradation rate.

The results of this combined approach pointed to a salt-mediated origin of the purple spots. The same approach, applied to severely damaged parchments (Migliore et al., 2019) highlighted again the presence of bacteriorhodopsin, and confirmed the “passage” of Halobacteria also by the NGS detection of their DNA in the purple spots. The LTA revealed that the deterioration had extended to the highly structured collagen fibers. In this case, due to the parchment conditions, the comparison was between severely and less damaged areas. The RAMAN spectra were also compared with the results obtained on conventionally cultured *Halobacterium* (Migliore et al., 2019). The presence of Halobacteriales in damaged parchments has been later confirmed also by Piñar et al. (2020).

Overall, this multidisciplinary approach yielded a great amount of simultaneous information, making possible to give a unitary reading framework to the various results obtained on purple spots over time.

The RAMAN spectrum detected bacterioruberin and other pigments, including the bacteriorhodopsin, a transmembrane, purple-pigmented proton pump associated with the cellular membrane of Halobacteria (Migliore et al., 2017, 2019), even when the DNA traces were absent. The NGS confirmed this result, blaming the halophilic archaea as the unequivocal culprit of the purple spot deterioration. LTA analyses revealed the different collagen populations attacked during the deterioration process, at the beginning the less structured *native* population, then even the highly structured *stabilized* ones. Furthermore, LTA depicted the overall degree of parchment degradation, highest in the highly damaged documents, as an extreme consequence of the profuse microbial attack on the collagen matrix.

In the light of these findings, Halobacteria can be charged of initiating the degradation process thanks to bacteriorhodopsin, which acts as a light-driven proton pump (Andersson et al., 2009) and converts light into chemical energy (ATP). This energy allows *H. salinarum* (i) to produce the proteolytic and lipolytic enzymes which attack and degrade the parchment collagen matrix (Bitlisli et al., 2004) and (ii) to feed on the amino acids present in the parchment (mainly arginine and aspartate), being amino acids the preferred source of chemical energy for Halobacteria (Ng et al., 2000).

This allowed to hypothesize that halophilic archaea are the powerful triggering actors, acting as a pioneer species in a two-phases heterotrophic microbial succession able to degrade the parchment (Migliore et al., 2017; Figure 1): the first phase is predictable, as mainly driven by rawhide salt-curing, which allows the halophilic and halotolerant microbes present in the marine salt to enter into the hides; it is common to all the damaged parchments, and triggered by Halobacteria that, in the appropriate humidity conditions, can grow and form the core of the purple spot damage, as it happens in brine-cured leathers (Bailey and Birbir, 1993; Perini et al., 2019; Enquahone et al., 2020). Differently, the second phase depends on the individual history of each parchment, which determines the identity of its colonizers. These last colonizers have great importance for Biocodicology, as they actually can give information on environment, manipulation, storage or even mishaps happened to the parchment during its history, although even the different haloarchaea may give information on the parchment homeland.

## Historical Perspective in the Study of Ancient Specimens

The NGS-based studies of ancient parchments (Migliore et al., 2016, 2017, 2019) allowed to hypothesize the history of their microbial colonizers, from the causal agent of the purple spots to the degradative dynamics of the microbial succession. However, the analyses of DNA extracted from ancient or historic specimens must take into account a fundamental and methodological problem, i.e., the small amount and the small size of DNA fragments from ancient microbial colonizers vs. the abundant and well-preserved DNA from the more recent parchment colonizers, as in the case of the analyzed parchment roll where no halobacterial DNA traces were found, although haloarchaeal pigments were unequivocally detected. Nevertheless, in the other ancient parchments analyzed (Migliore et al., 2019; Perini et al., 2019; Piñar et al., 2020) the 16S rDNA-targeted metagenomics showed the presence of haloarchaea DNA strands long enough to allow their taxonomic identification, bypassing the burdensome issues of the study of ancient DNA small fragments.

Anyway, the studies of ancient parchments must always take into consideration the possible limited availability of the oldest DNA strands while evaluating the colonization process on an historical perspective. In fact, the analysis of metagenome provides a flattened picture, a two-dimensional representation of the colonization process that, instead, occurred on a three-dimensional perspective, which include the stratification of generations of different microbes succeeded over centuries. The most recent and environmental readily available colonizers are expected to be quantitatively exceeding the old ones and are easily detectable due to the good conservation status of DNA. Bacteria, mainly Actinobacteria (e.g., *Saccharopolyspora* spp.) and fungi (*Penicillium* and *Aspergillus* among others) dominate the documents. Their being more recent can be hypothesized not only by the relative DNA abundance, but also by the presence of apparently integer cells and spores (SEM, Piñar et al., 2015, 2020), and the ability to long standing dryness.

To have a clear experimental evidence of parchment colonization by haloarchaea and overcome the limits of the historical perspective in studying ancient specimens, the case of hide-products biodeterioration was investigated with a “modern” perspective, namely by analyzing present-day damaged leather products. In these studies, both the raw marine salt and the salted rawhides, to be used in tannery, contained cultivable haloarchaea (Perini et al., 2019; Enquahone et al., 2020) and tanned leathers—showing signs of biodeterioration similar to purple spots, *a.k.a.* red heat deterioration—showed the signature of haloarchaeal DNA.

The same multidisciplinary approach used in the previous studies, applied to damaged vs. undamaged samples of Chrome(III) tanned leathers, unequivocally demonstrated that the microbial culprits of present-day hide-products belong to the Halobacteriaceae family, mainly *Halobacterium* and *Halorubrum* (Perini et al., 2019). Halophilic microbes associated with red heat damage in salted sheepskin demonstrated to be highly diverse: they depend on the sea-salt used for rawhide salt-curing, which contains the halophilic and halotolerant microorganisms (archaea, marine bacteria, and their resting phases) living into the evaporation ponds of salt-fields. Enquahone et al. (2020) found into nowadays salt-treated hides a different species of Halobacteriales, the haloarchaea *Halococcus*, although colonization process and effects are comparable to those found in Italian rawhides. The difference surely depends on the different region of salt production, being the rawhide studied in Addis Ababa, Ethiopia. Nevertheless, in the hides intended for commercial use the preventive measures targeting such group of halophilic microbes seem unnecessary. In fact, tanned leather manufacturing includes a series of treatments, such as pickling, liming, tanning, drying, dyeing, trimming, finishing, and Chrome(III) treatment, which make highly toxic the environment within the hides. These treatments have the double effect of killing all the microbes present in the rawhide and stabilizing the collagen matrix (Perini et al., 2019) producing new chemical bonds (Kite and Thomson, 2006), even in already colonized and damaged areas.

**FIGURE 2:**
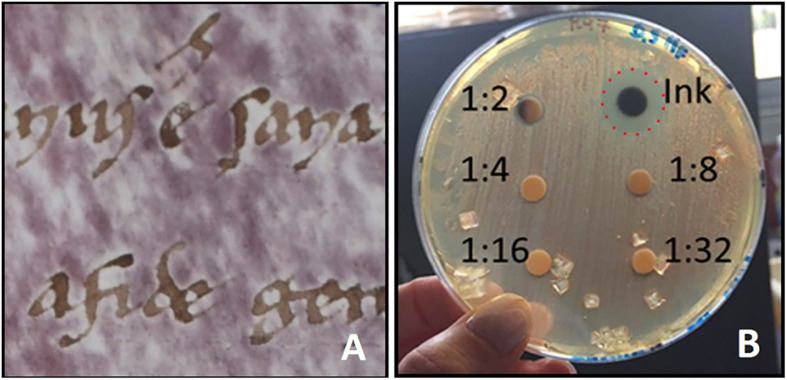
**(A)** “Halo of inhibition” of purple spots around ink letters on a written parchment [from Vendittozzi (2015)]; **(B)** A toxicity test shows the inhibitive effect of ion-gallic ink on the growth of *Halobacterium salinarum* on selective solid medium M97 [from Perini (2019)]. The ion-gallic ink was made with FeSO_4_⋅7H_2_O (25.53 g/L), CuSO_4_⋅5H_2_O (15.41 g/L), tannic acid (49.20 g/L), acacia gum (31.40 g/L); the final solution was twofold diluted (1:2) with distilled water. The reference strain *H. salinarum* DSM 3754 was grown at 37°C for 7 days on liquid media M97; the microbial suspension was adjusted to 0.5 McFarland standard (O.D._600__nm_ = 0.14); 100 μl of the microbial suspension were inoculated on solid medium M97. Successively, six sterile filter paper disks of 5 mm diameter were located on the medium surface. Twofold serial dilutions (1:2, 1:4; 1:8, 1:16, and 1:32) of the ion-gallic ink were prepared and 10 μl of each dilution were dropped on the corresponding disk. Plate was incubated at 37°C for 7 days. The ion-gallic ink, containing high amounts of copper and iron, is able to inhibit *H. salinarum* growth (red dashed line); whilst, the other dilutions did not show antimicrobial effects.

Conversely, preventive measures of parchment contamination could have been useful in the past to avoid parchment decay: in fact, parchment manufacturing included only “mild” treatments and mechanical removal of skin layers (Guareschi, 1905) which just eliminate the more external layers of the colonized rawhide but do not kill or remove the microbes deep within the hide, and do not affect the collagen matrix structure and stability. Moreover, the reviving of halobacteria has been possible over centuries, and surely occurred after the parchment writing and decoration, as demonstrated by the lack of purple spots around the letters (Piñar et al., 2015; Vendittozzi, 2015; Figure 2A) due to the toxic components of ancient inks which inhibited halobacterial growth. For example, the ion-gallic ink, the “common ink” used to write manuscripts from the Middle Ages to the 19th century (Ceres, 2007; Kolar et al., 2008), includes high amounts of copper and iron, that elicit inhibiting effect on halobacterial growth (Perini, 2019; Figure 2B).

## Conclusion and Perspectives

The metagenomic approach allowed to understand, among other, that ancient parchments are extreme environments, harboring purple spots producing Halobacteria (Migliore et al., 2017, 2019). Cultural heritage manufacts have been never considered extreme environments, and hardly they can be considered such. Hence, the metagenomic approach changed in a definitive way our perception of these ancient manufacts, which contain a great deal of our cultural heritage, but also it opened a new perspective on the studies of other cultural heritage manufacts, which can be now considered as extreme environments either, where halophilic and halotolerant microorganisms can thrive, producing damages (Perini, 2019). In fact, not only parchment but also other culturally important materials (buildings, sculptures, and artworks) are saline, or can become such. Salt can enter into building materials as gypsum (calcium sulfate, CaSO_4_) or alkali sulfates, but building materials can be infiltrated by salt from the environment through capillary force, as in the case of caves, or the buildings that receive inputs of salt water from natural sources (as seawater spray), or when salts are used for de-icing.

Hence, metagenomics may help answer relevant questions and contribute to a better understanding of the history of ancient manufacts, other than ancient parchment manuscripts. In the present-day research *scenario*, metagenomics offers a powerful approach to characterize the complex (mainly not-cultivable) microbial communities that colonized the ancient documents over time. Being able to capture the complexity of biodeterioration, metagenomics conquers a place on the podium among other molecular approaches because it maximizes the understanding of the microbial composition and helps in deciphering the dynamics of biodeteriogen communities. In the case of limited availability of the oldest DNA strands shotgun metagenomics may face the problem, allowing to check for the presence of fragments of different size, including the short damaged ones. On the contrary, proteomics seems less useful in the case of parchments due to the intrinsic protein composition of the matrix, which may overcome all the other signals, and metabolomics could be unable to traces metabolites produced langsyne and subjected to further colonization.

Anyway, the concurrent use of other “omics” may help to understand biology at the aggregate level, enabling the analysis of both the whole microbial structure and how microbes might influence each other’s activities affecting collective functions. However, only the integration of many different techniques (physical, chemical, growth-dependent and independent microbiological techniques, and different “omics”) allows to produce a comprehensive analysis of the biodeterioration of cultural heritage goods (Marvasi et al., 2019). In any case, the taxonomic identification may give some insights into the potential microbial functionality, but not on the biochemical processes, due the high metabolic versatility of microbes which allows different microbes to play the same functional role. To this end metatrascriptomics, which allows to obtain the whole gene expression profiling of a microbial community based on next-generation and third-generation sequencing, may help to solve this issue. However, the use of this and other “omics” must always take into consideration the flattened historical perspective which limits the identification of previous processes, putting the spotlight on the more recent colonization events. Nevertheless, over time many studies showed the benefits associated with the combination of approaches (Gutarowska et al., 2015; Migliore et al., 2017, 2019; Adamiak et al., 2018), gathering a lot of new information useful to develop intervention strategies for the preservation of cultural heritage artifacts.

In conclusion, the use of multiple approaches including the omics techniques, will allow an unprecedented level of knowledge on the biological damage and its dynamics, and could be applied to different cultural heritage manufacts. The high-throughput techniques would allow the structural and functional characterization of the biodeteriogen microbial communities, by the sequencing/identification of total pools of biomolecules including DNAs, proteins, or metabolites. As regard the parchments, this information will allow to keep preserved the beauty of these timeless masterpieces of our cultural heritage.

## Author Contributions

NP and LM conceived the manuscript. NP prepared a first draft of the manuscript, with support from FM and SO. LM and MT edited and reviewed the draft to obtain the final manuscript, which has been discussed and approved by all the authors.

## Conflict of Interest

The authors declare that the research was conducted in the absence of any commercial or financial relationships that could be construed as a potential conflict of interest.
